# Association of Anemia With Epilepsy and Antiepileptic Drugs

**DOI:** 10.7759/cureus.19334

**Published:** 2021-11-07

**Authors:** Jaskamal Padda, Khizer Khalid, Mohammad Syam, Varsha Kakani, Gauvain Kankeu Tonpouwo, Richa Dhakal, Sandeep Padda, Ayden Charlene Cooper, Gutteridge Jean-Charles

**Affiliations:** 1 Internal Medicine, JC Medical Center, Orlando, USA; 2 Internal Medicine, AdventHealth and Orlando Health Hospital, Orlando, USA

**Keywords:** epilepsy, antiepileptic drugs, anemia, seizures, aplastic anemia, hemolytic anemia, megaloblastic anemia

## Abstract

Epilepsy is a disorder that causes unprovoked seizures regularly. It affects between 1% and 3% of the population. After the first seizure, the chances of having another one are almost 40%-52%. The etiology of febrile seizures in children with sickle cell disease is still unknown. In some groups, iron deficiency anemia has been linked to an increased risk of seizures. Although the reason and process are uncertain, some people believe that taking iron supplements can help prevent seizures. This literature covers haptene, non-haptene immune-related hemolysis, and oxidative processes activated by anti-seizure medications (ASMs). In epileptic patients, ASMs can cause anemia. Folic acid can be given to carbamazepine-treated anemic patients. There is growing evidence that it improves hemoglobin and leukocytes in individuals who take it. Therefore, one of the most efficient strategies to avoid future seizures is to take ASMs daily to maintain an even level of anticonvulsant in the body. To prevent further seizures, lifestyle changes are essential. Further studies and clinical trials are warranted to prove a clear association between epilepsy and hematologic disease, which will improve quality of life in the future.

## Introduction and background

Seizures are one of the most common neurological disorders [1]. It is estimated that approximately 10% of the population experience one or more episodes of seizures at least once in their lifetime [1]. A seizure is a paroxysmal alteration in brain function caused by excessive discharge from the neurons in the brain [2]. Epilepsy is a condition that results in recurrent unprovoked seizures. It occurs in approximately 1%-3% of the population [1]. There are differences in the incidence of epilepsy from one geographical location to the other. Few regions like Norway and England show a low incidence of 16 to 47 per 100,000 population, while few regions like rural China demonstrate a high incidence rate of 111 per 100,000 population [3]. Approximately 2%-5% of the children in the age group of 3-60 months are affected by febrile seizures [4]. Some studies show that iron deficiency anemia can be a risk factor for the occurrence of febrile seizures in children [4]. However, few studies also show that iron deficiency acts as a protective factor against seizures by increasing the seizure threshold [5]. Another association between epilepsy and anemia occurs in cases where anemia is caused as a side effect of anti-seizure medications (ASMs). These drugs are associated with a range of hematologic disorders ranging from mild thrombocytopenia or neutropenia to severe anemia, red cell aplasia, or bone marrow failure. The exact mechanism of this effect is not known but is considered to be due to immune-mediated mechanisms and the pharmacokinetic and pharmacodynamic interaction of the drugs [6]. Thus, a proper relationship between anemia, epilepsy, and ASMs is necessary for a physician to keep an eye on the blood counts while treating patients with epilepsy [7]. In this literature, we will discuss the association between anemia and epilepsy, the pathophysiology of hematologic diseases by ASMs, along with management and prevention of seizures.

## Review

Epilepsy

The practical definition of epilepsy is the occurrence of one episode of unprovoked seizure with a high risk of another episode [8]. The risk of an episode of seizure after the first episode is nearly 40%-52%, and after two unprovoked afebrile seizures, the four-year risk is 73% [8]. Factors provoking seizures include head trauma, inflammatory brain injury, infections of the central nervous system, stroke, neoplasms of the central nervous system, and neurodegenerative disorders such as Alzheimer's disease in adults [3]. Classification of epilepsy also depends on many factors such as family history, age of onset, electroencephalogram, and neuroimaging. This classification helps the physician discuss the natural course of the disease and its prognosis [1]. The newer and the latest standard of classification of epilepsies is the 2017 International League Against Epilepsy (ILAE) seizure classification. This classification presents at three levels considering etiology at each level. Level 1 involves the diagnosis of seizure type, which is mostly assumed to be epileptic. After the diagnosis of seizure type is made, the second level involves the epilepsy type. Epilepsy type can be either focal generalized, generalized, combined generalized, and focal or unknown type. The third level is that of epilepsy syndrome where the specific syndromic determination is made [9]. Seizures may also be induced because of secondary causes like hypoglycemia. These are provoked seizures and are relieved by treating the provoking factors. ASMs do not have a role in the treatment of such conditions [1,2]. Treatment of unprovoked seizures includes ASMs. The selection of appropriate ASMs depends on the pharmacokinetics of the drug, side effects, and cost [1]. Refractory epilepsy, which constitutes one-third of the cases of epilepsy, is defined as the seizures not controlled by two or more ASMs or other therapies [2].

The effect of anemia on epilepsy

Epilepsy in sickle cell anemia is two to three times more common than in the general population. It is also related to an increased rate of early death in children. A febrile seizure is one of the most prevalent causes of seizure in children with sickle cell disease, especially in malaria-endemic areas [10]. In adolescents and young adults with sickle cell disease, epileptic seizures and paraplegia are more common [11]. Also, in some younger populations, most patients with epilepsy were found to have a prior history of stroke [12]. According to the observations in a study, it is believed that vasculopathy and localized hypoperfusion may play a role in the progression of seizures in sickle cell disease [13]. However, the cause of febrile seizures in children with sickle cell disease is not fully understood yet.

In some studies, iron deficiency anemia increases the risk of febrile seizures in children, probably because it is one of the essential factors for growth, development, and immunity [14]. In addition, anemia reduces the seizure threshold, resulting in increased seizure activity. On the other hand, some studies believed it to be a protective factor [4]. Anemia can cause seizures through various processes, including a drop in gamma-aminobutyric acid inhibitory neurotransmitters, alterations in neuron metabolism, enzyme reduction, and a reduction in brain oxygenation and energy metabolism. Although the cause and mechanism are not fully understood, some of the authors believe that iron supplementation might decrease the incidence of seizures in some populations [15].

Pathophysiology of hematologic diseases by ASMs

Anemia is one of many adverse effects induced by ASMs. According to the World Health Organization classification of adverse effects, anemia influenced by ASM corresponds to type B and type C effects, which are idiosyncratic and chronic effects, respectively [16]. Type B effects are direct cytotoxic or immunologic reactions induced by ASM or their metabolites, whereas type C effects refer to the ASMs cumulative dose effect [17]. Different types of anemia have been described following the use of ASM and each class is related to specific drugs and underlined by precise mechanisms.

Aplastic anemia

Idiosyncrasies induced by the drug itself or its metabolites in the context of aplastic anemia are mainly non-immune-mediated. Aplastic anemia, in this case, is related to the direct toxicity of bone marrow cells by the ASM or its metabolites. An example here is felbamate, an ASM used for secondarily generalized seizures in patients refractory to other agents [18]. Felbamate-induced bone marrow suppression is explained by its two active metabolites, atropaldehyde and alcohol carbamate. Once they are synthesized in the liver, they will bind albumin and be transported to the bone marrow where they induce direct cellular damage [19]. Another ASM causing non-immune aplastic anemia is carbamazepine. It is metabolized through oxidative pathways in the liver, leading to toxic aromatic metabolites, such as carbamazepine 10,11-oxide. These metabolites will induce direct damage to the bone marrow's erythroid progenitors, leading to aplastic anemia [20]. A few cases of aplastic anemia were described as well with phenytoin, valproate, and ethosuximide through interference with deoxyribonucleic acid (DNA) synthesis and covalent binding to erythroid progenitor macromolecules, thus inducing direct cytotoxicity [6].

Hemolytic anemia

Idiosyncrasies induced by ASM in the frame of hemolytic anemia are both immune and immune-mediated. Haptene, non-haptene immune-related hemolysis, and oxidative reactions are described here [21]. Immunologic hemolysis here is under type II hypersensitivity [22]. In heptane reactions, the ASM binds tightly to one of the erythrocyte's membrane peptides, the complex will then be identified by the adaptive immune system as foreign, further inducing antibodies by activated B cells hemolysis.

In non-haptene reactions, the ASM binds loosely to the red blood cell membrane, inducing the production of antibodies that coat the cell and lead to either activation of the complement system with subsequent hemolysis or activation of the natural killer cells (antibody-dependent cellular cytotoxicity) [21,22]. One case of hemolytic anemia induced by oxcarbazepine in a 75-year-old male was reported by Chaudhry et al. [21]. Another mechanism involved in hemolytic anemia induced by ASM is oxidative injury. Certain ASM metabolites induce erythrocyte membrane lipid peroxidation, making it susceptible to hemolysis. The most vulnerable patients are those with red blood cell enzyme altered activity [21]. Yamamoto et al. described this in a five-year-old boy with an underlying personal history of reduced glutathione peroxidase activity who presented with intravascular hemolytic anemia five weeks following administration of carbamazepine. The patient clinically improved after drug discontinuation [20].

Megaloblastic anemia

Megaloblastic anemia is a type C ASMs' adverse effect related to cumulative dose effect, which is consecutive to chronic drug use. Several mechanisms have been identified as prevailing regarding folate deficiency associated with long-term usage of certain ASMs. These include ASM-induced folic acid conjugase inhibition leading to impaired folate absorption; other mechanisms include increased folate coenzyme demand, hepatic ASM detoxification, and ASM-induced folate displacement from serum carrier proteins [23].

A case-control study regarding folic acid levels in epileptic patients on carbamazepine, oxcarbazepine, or valproate led by Aslan et al. showed a decrease in the mean folic acid level as well as a marginal level of folic acid in 13.2% of patients and a deficient level in 4.4% of patients [23]. Another contributing factor to megaloblastic anemia is the decreased level of vitamin B12 correlated to the prolonged intake of certain ASM such as pregabalin, topiramate, and primidone. This observation was sustained and corroborated by Linnebank et al. in a prospective monocentric study aiming to test ASM-associated vitamin B12 deficiency [24].

Management

In a patient with anemia, the general causes should be ruled out first. It could be due to some underlying conditions (vitamin or mineral deficiency, anemia, inflammation, bleeding, malignancy, etc.) or any other exposures such as drugs. Among ASMs, anemia is more common with the use of older medications compared to the newer ones in epileptic patients [6]. Therefore, during ASM therapy, routine blood count monitoring is beneficial and should be done [23]. In addition, homocysteine, vitamin B12, folic acid levels, and blood smears should also be routinely evaluated, as abnormal values have frequently been reported [23]. These parameters help us monitor the condition and act promptly if any abnormalities are noted.

Folic acid can be used in anemic patients under carbamazepine. Though the exact mechanism of action of folic acid is not known yet, there has been increasing evidence to show improvement in hemoglobin and leukocytes in patients using this drug. However, more research is needed to determine the ideal dose for maximizing the benefits of folic acid [25].

A study showed that phenytoin and carbamazepine cause toxicity in erythroid precursor cells due to defects in arene oxide drug metabolite detoxification, reducing DNA synthesis [26]. In contrast, another study believed drug-induced toxicity caused by selective inhibition of DNA synthesis in erythroid precursors, most likely at the deoxyribose production phase, leads to aplastic anemia [27]. This is managed by using IV human recombinant granulocyte-macrophage colony-stimulating factor [28]. Also, drugs like phenytoin and carbamazepine should be avoided in patients with porphyria as they may lead to hemolysis. In other drugs, where there is anemia due to folate deficiency, dietary and oral supplementation can reverse the condition [6].

In the case of drug-induced hemolysis, particularly with drugs like oxcarbazepine, the primary step in management is to hold the drug. Then, we should look at the lab values and assess the patient for necessary transfusion [21]. In sickle cell anemia-induced epilepsy, a pediatric neurologist should be consulted to rule out other neurological complications like stroke, transient ischemic attack, or migraine. If specialist care is not available, then a skilled pediatrician should be consulted for a thorough neurological examination [9].

Prevention

**Figure 1:**
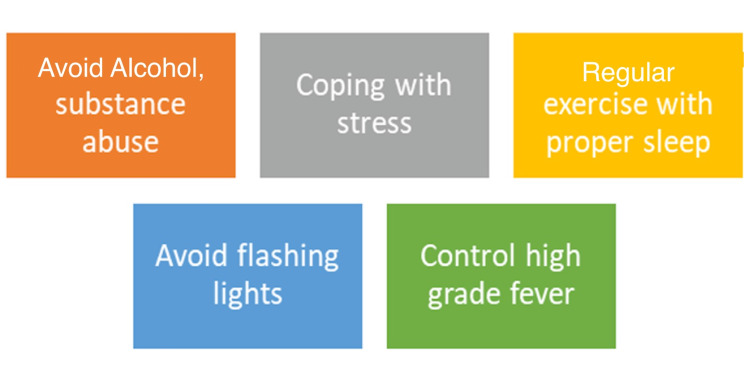
Epilepsy seizure prevention. The recurrence of seizures can be reduced tremendously via lifestyle/environmental modifications [30]. Image created by the author (Mohammad Syam, MBBS, MPH).

Preserving brain function along with minimizing the risk of injuries and death are crucial steps in seizure prevention [29]. It is also based on the overall management and treatment strategies, including compliance to medication for uncontrolled seizures. Additionally, lifestyle and environmental modifications have a tremendous impact on reducing seizure recurrence (Figure 1) [30].

One of the most efficient strategies to avoid future seizures is to take ASMs daily to maintain an even level of anticonvulsant in the body. ASMs should be used carefully to reduce bone abnormalities such as bone thinning or osteoporosis and reproductive or hormonal imbalances that affect fertility, along with cognitive and coordination impairments [30]. Drug-associated hematologic illness has been reduced when novel ASM, including gabapentin, lamotrigine, and topiramate, have been used [31]. Hence, supplementing with folic acid and monitoring blood counts regularly should be the mainstay in preventing anemia caused by ASM. Furthermore, advent treatment modalities consist of a vagus nerve stimulator, and surgical options such as laser ablation and nerve responsive neurostimulator insertion are currently being practiced for recurrence. These new promising therapeutic options may provide additional preventive opportunities for all ages of life [31].

## Conclusions

Seizures are characterized by the excessive discharge from the neurons in the brain and are estimated to occur in approximately 10% of the population at least once in their lifetime. Epilepsy is the term used for unprovoked seizure activity and has various causes, which can be idiopathic or symptomatic. A higher incidence of epilepsy was seen in patients with sickle cell disease than in the general population. Studies show contradictory results regarding the association of febrile seizures in children with iron deficiency anemia; few studies show it as a protective factor, while others show it as a risk factor. ASM forms the mainstay of treatment in epilepsy, but ASMs have many side effects, the main one being anemia. Lifestyle modifications have been shown to prevent further episodes of seizures. Regular monitoring of blood reports and supplementation with folic acid may also prevent severe anemia in patients on ASMs. Further research is required on ways to overcome the side effects of ASMs and find the appropriate dose of folate supplementation that will prevent the development of anemia.
